# A New Dimension to Ras Function: A Novel Role for Nucleotide-Free Ras in Class II Phosphatidylinositol 3-Kinase Beta (PI3KC2β) Regulation

**DOI:** 10.1371/journal.pone.0045360

**Published:** 2012-09-13

**Authors:** Katy A. Wong, Angela Russo, Xuerong Wang, Yun-Ju Chen, Arnon Lavie, John P. O'Bryan

**Affiliations:** 1 Department of Pharmacology, University of Illinois College of Medicine, Chicago, Illinois, United States of America; 2 Cancer Center, University of Illinois College of Medicine, Chicago, Illinois, United States of America; 3 Center for Cardiovascular Research, University of Illinois College of Medicine, Chicago, Illinois, United States of America; 4 Biochemistry and Molecular Genetics, University of Illinois College of Medicine, Chicago, Illinois, United States of America; Children's Hospital Boston & Harvard Medical School, United States of America

## Abstract

The intersectin 1 (ITSN1) scaffold stimulates Ras activation on endocytic vesicles without activating classic Ras effectors. The identification of Class II phosphatidylinositol 3-kinase beta, PI3KC2β, as an ITSN1 target on vesicles and the presence of a Ras binding domain (RBD) in PI3KC2β suggests a role for Ras in PI3KC2β activation. Here, we demonstrate that nucleotide-free Ras negatively regulates PI3KC2β activity. PI3KC2β preferentially interacts *in vivo* with dominant-negative (DN) Ras, which possesses a low affinity for nucleotides. PI3KC2β interaction with DN Ras is disrupted by switch 1 domain mutations in Ras as well as RBD mutations in PI3KC2β. Using purified proteins, we demonstrate that the PI3KC2β-RBD directly binds nucleotide-free Ras *in vitro* and that this interaction is not disrupted by nucleotide addition. Finally, nucleotide-free Ras but not GTP-loaded Ras inhibits PI3KC2β lipid kinase activity *in vitro*. Our findings indicate that PI3KC2β interacts with and is regulated by nucleotide-free Ras. These data suggest a novel role for nucleotide-free Ras in cell signaling in which PI3KC2β stabilizes nucleotide-free Ras and that interaction of Ras and PI3KC2β mutually inhibit one another.

## Introduction

Ras is a monomeric G-protein that cycles between GDP- and GTP-bound states [1]. The nucleotide state of Ras is tightly regulated *in vivo* by two classes of proteins: guanine nucleotide exchange factors (GEFs) and GTPase activating proteins (GAPs). GEFs facilitate the release of bound nucleotide from Ras to produce nucleotide-free Ras which preferentially binds GTP due to the higher cellular concentrations of GTP over GDP thereby leading to Ras activation. In contrast, GAPs enhance the intrinsic GTPase activity of Ras to facilitate the hydrolysis of GTP to GDP resulting in Ras inactivation. Mutations in the Ras genes (H-, K-, and N-Ras) are found in approximately 30% of human tumors [1]. These mutations typically impair the GTPase activity leading to elevated RasGTP levels and aberrant cell growth due to the chronic engagement of Ras effectors.

RasGTP has been considered to be the only biologically active form of Ras, although accumulating evidence lends support to the contrary [2], [3], [4], [5]. Over-expression of wild-type Ras, which is thought to be predominantly GDP-bound due to its intrinsic GTPase activity, exerts a suppressive effect on oncogenic transformation by constitutively activated Ras alleles [2], [3], [4], [5]. In a chemical carcinogenesis model for lung tumorigenesis, mice hemizygous for K-Ras deletion develop 4–5 times more lung tumors than wild-type mice suggesting that the wild-type allele plays a protective role [2]. A number of human tumors exhibit loss of heterozygosity of K-Ras further supporting the notion that wild-type Ras functions as a tumor suppressor [6], [7], [8]. However, these studies do not distinguish whether the suppressive effect of wild-type Ras is due to higher levels of RasGDP or potentially nucleotide-free Ras. Indeed, a similar oncosuppressive effect is seen with dominant-negative Ras, Ras17N, which exerts its inhibitory effect by competing for GEFs due to its low affinity for nucleotides (compared to wild-type Ras) and therefore longer lifetime in the nucleotide-free state [9]. However, the oncosuppressive effect of Ras17N on Ras-mediated transformation is independent of its interaction with exchange factors suggesting a potential role for nucleotide-free Ras in cellular signaling [10], [11], [12].

The above findings lead to the question of whether Ras proteins play a role in signaling independent of nucleotide binding. Indeed, our studies on intersectin1 (ITSN1) provide and example of such a role. ITSNs are multi-domain scaffolding proteins that regulate multiple biochemical pathways in addition to playing a central role in endocytosis [13]. There are two ITSN genes, ITSN1 and ITSN2, each coding for a short (S) and long (L) isoform [14]. The short isoform contains two Eps15 homology (EH) domains, followed by a coiled-coiled (CC) domain, and five Src homology 3 (SH3) domains [14], [15], [16], [17]. The longer isoform contains an additional Dbl homology (DH) domain, pleckstrin homology (PH) domain, and C2 domain [18]. ITSN1 was initially discovered as a regulator of endocytosis, but recent studies have uncovered a role for this adaptor in intracellular signaling pathways including kinase activation, receptor tyrosine kinase regulation, and compartmentalized-specific Ras activation [15], [19], [20], [21], [22], [23], [24]. We previously reported that ITSN1-S stimulates H-Ras (hereafter referred to as Ras) activation on intracellular vesicles [24]. However, the target of this ITSN1-Ras pathway was unclear. The role of ITSN1 in Ras and PI3KC2β regulation combined with the presence of a putative RBD on PI3KC2β lead to the hypothesis that Ras is involved in ITSN1 regulation of PI3KC2β activation. Indeed, our results reveal a unique role for nucleotide-free Ras in negatively regulating PI3KC2β activity suggesting a potential broader role for this form of Ras than previously recognized.

**Figure 1:**
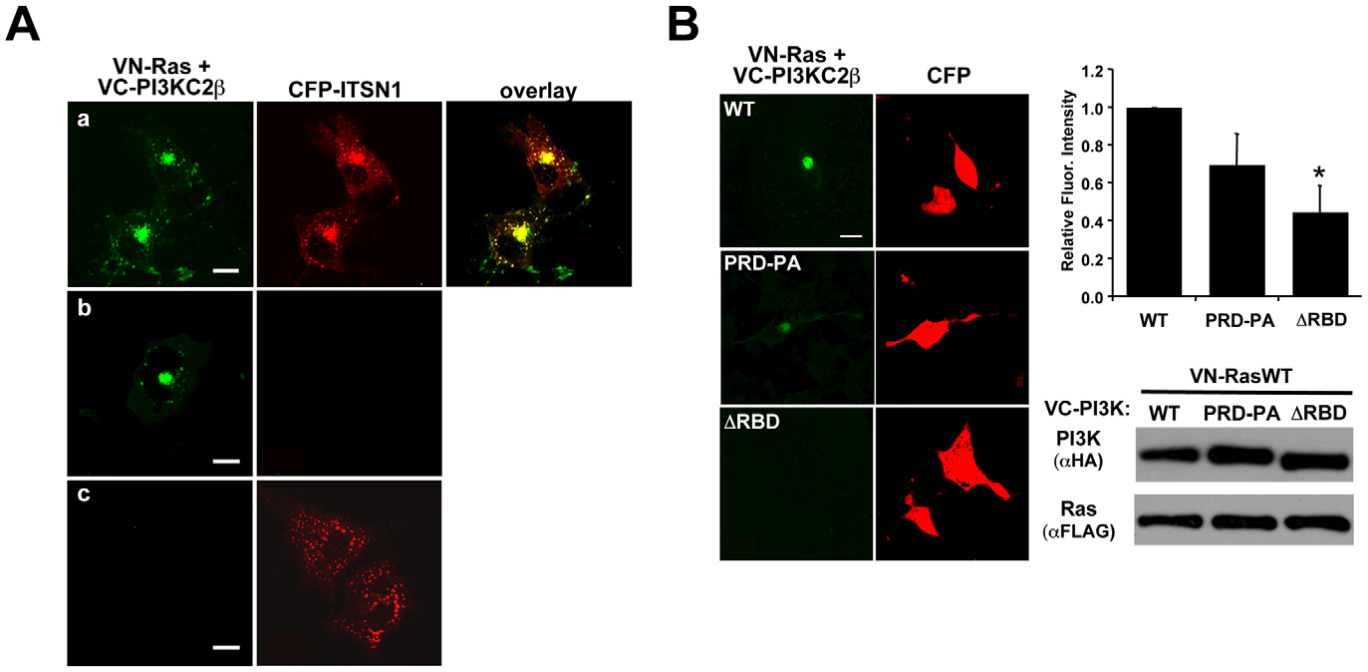
Ras, PI3KC2β, and ITSN1 co-localize on intracellular vesicles. (A) VN-Ras and VC-PI3KC2β (green) were co-transfected with CFP-ITSN1 (red) into COS cells. a. The PI3KC2β-Ras BiFC complex co-localizes with ITSN, represented by yellow in the overlay panel; b. The VN-Ras and VC-PI3KC2β YFP signal (green) does not bleed into the CFP channel; c. The CFP-ITSN1 signal does not bleed into the YFP channel (size bars  = 20 μm). Note: the differences in signal strength of the BiFC signal in (a) vs (b) are due to a lower power setting for the laser in (b) so that pixel intensities can be accurately quantified and are not saturated. In (a) a higher laser power was used to illustrate the punctate localization of the Ras-PI3KC2β complex throughout the cell. (B) Ras interaction with PI3KC2β is disrupted by deletion of the RBD (ΔRBD) but not by mutation of the Pro-rich, ITSN1 binding sites (PRD-PA). The graph represents the average fluorescence intensity per cell ± S.E.M. from at least three independent experiments (*p<0.05, WT vs ΔRBD, PRD-PA vs ΔRBD). Western blot analysis demonstrates equal expression of all constructs (size bars  = 20 μm).

**Figure 2:**
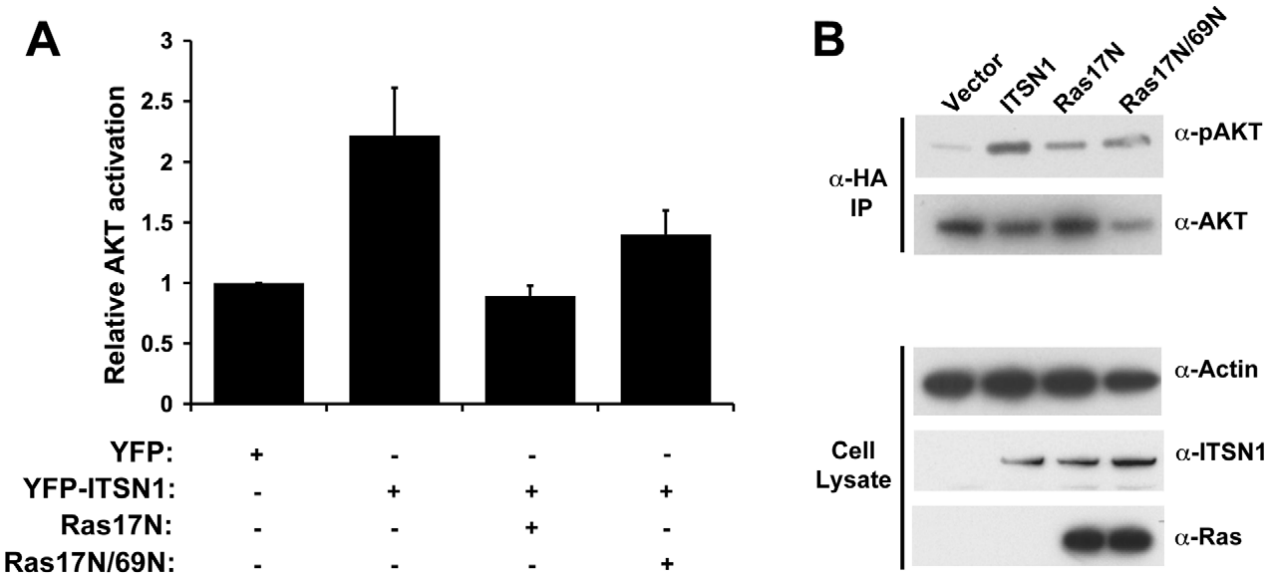
Ras is necessary for ITSN1 activation of AKT. (A) YFP-ITSN1 overexpression stimulates AKT activation as measured by levels of phospho-AKT as previously described [22]. Co-expression of Ras17N or Ras17N/69N inhibits this response. Results represent the average fold activation of AKT ± S.E.M. from at least three independent experiments. (B) Western blot analysis of AKT activation from a representative experiment. Top two panels represent Western blots of HA immunoprecipitates of cell lysates to assess AKT activation as described in the Materials and Methods section. The lower three panels indicate the level of expression of ITSN1, Ras, and actin (a loading control).

## Materials and Methods

### Cell lines, transfection and reagents

COS cells (kindly provided by Dr. John Cidlowski, NIH) [21] were maintained in Dulbecco's Modified Eagle Medium (DMEM) supplemented with 10% fetal bovine serum. COS Cells were transfected with Lipofectamine (Invitrogen, Carlsbad, CA) as recommended by the manufacturer. Antibodies used include anti-hemagglutinin (HA) (Covance, Emeryville, CA), anti-Flag (Sigma, St. Louis, Missouri), anti-GST conjugated to HRP (Santa Cruz Biotechnology, Santa Cruz, CA), anti-Ras (Santa Cruz Biotechnology, Santa Cruz, CA), anti-Rab5 (Santa Cruz Biotechnology, Santa Cruz, CA), anti-AKT (Cell Signaling Technology, Danvers, MA), anti-AKT-p473 (Cell Signaling Technology, Danvers, MA).

**Figure 3:**
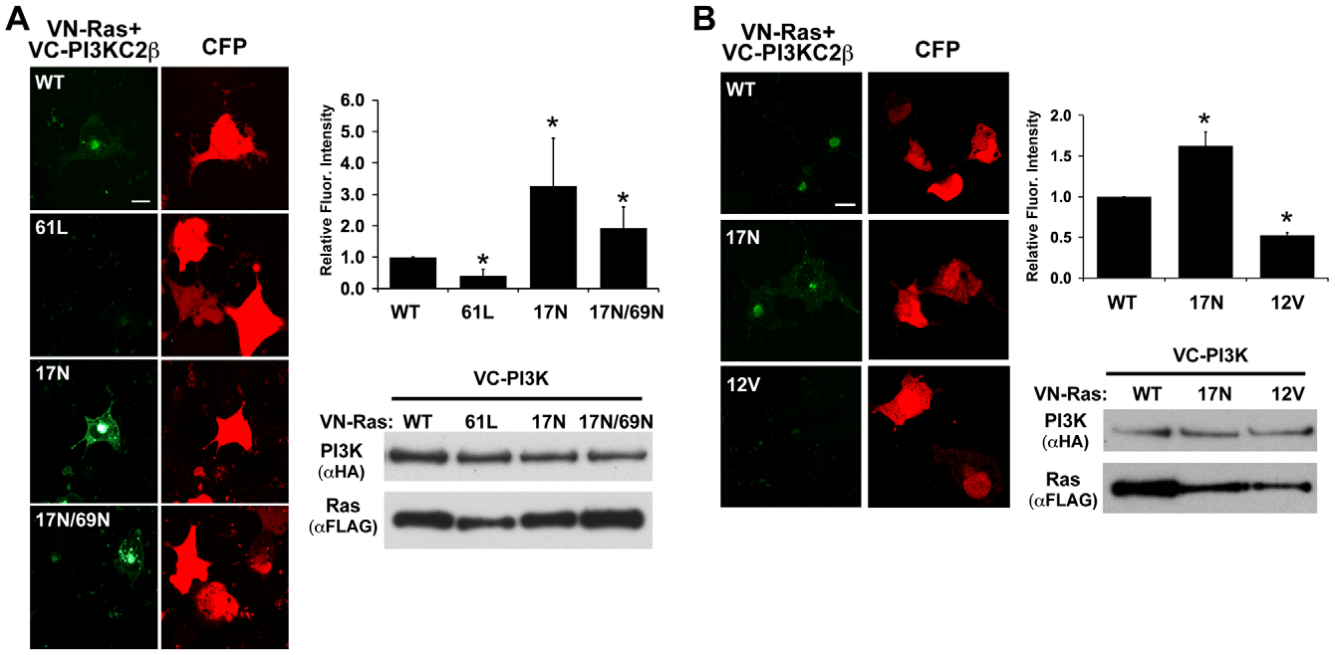
Ras forms a complex with PI3KC2β. (A) PI3KC2β preferentially interacts with Ras17N. VC-tagged PI3KC2β was co-transfected with one of the following VN-tagged Ras constructs: WT, 61L, 17N, or 17N/69N. BiFC signal (green) demonstrates that PI3KC2β interacted with Ras17N >17N/69N >WT >61L. CFP (red) was used as a transfection control. The graph represents the average fluorescence intensity per cell ± S.E.M. from at least three independent (*p<0.05). Western blot analysis demonstrates equivalent expression of all constructs. (B) PI3KC2β does not interact with Ras12V. VC-tagged PI3KC2β was co-transfected with one of the following VN-tagged Ras constructs: WT,17N, or 12V. BiFC signal (green) demonstrates that PI3KC2β interacted with Ras17N >WT >12V. CFP (red) was used as a transfection control. The graph represents the average fluorescence intensity per cell ± S.E.M. from at least three independent experiments (*p<0.05). Western blot analysis demonstrates expression of all constructs (size bar  = 20 μm).

**Figure 4:**
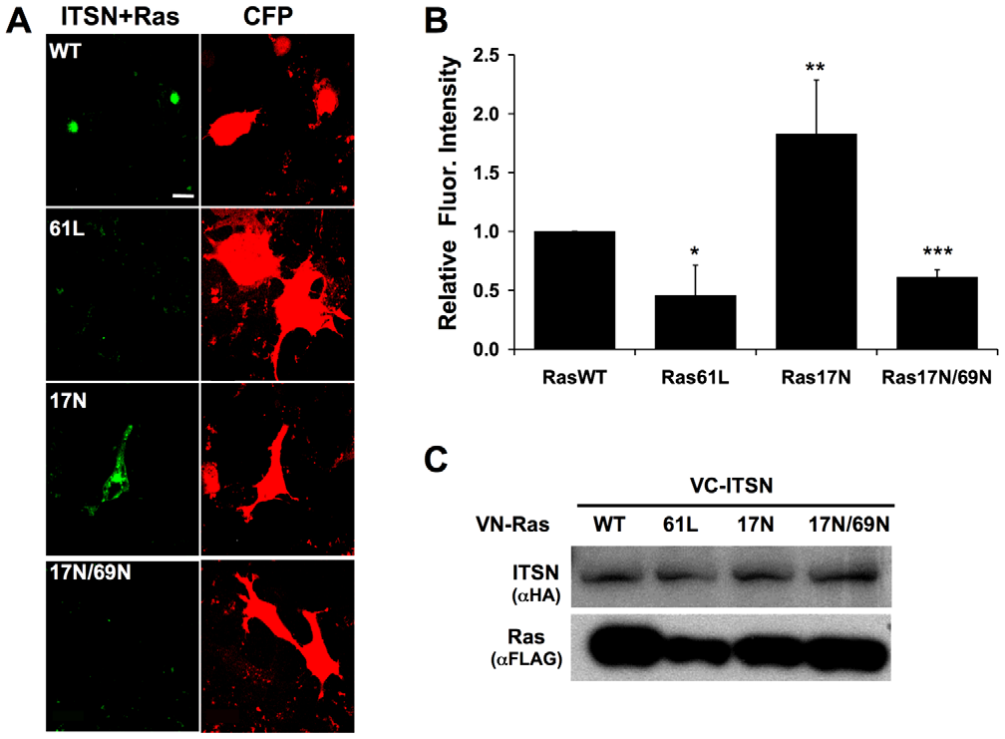
ITSN1 and Ras form a BiFC complex. (A) VC-tagged ITSN1 was co-transfected with one of the following VN-tagged Ras constructs: WT, 61L, 17N, or 17N/69N. ITSN1 formed a complex (green) with Ras17N >WT >17N69N >61L. CFP (red) was used as a transfection control. (B) The graph represents the average fluorescence intensity per cell ± S.E.M. from at least three independent experiments (*p<0.05, **p<0.01). (C) A Western blot was performed to demonstrate equal expression of all constructs. (size bar  = 20 μm).

### DNA constructs and generation of mutants

The BiFC vectors pFlag-VN-173N and pHA-VC155N were gifts from Dr. Chang-Deng Hu (Purdue University, West Lafayette, IN). CFP-PI3K was cut with XhoI, blunt ended with Klenow, digested with KpnI, and then cloned into the pHA-VC155N vector that had been cut with EcoRI, blunted ended with Klenow, and then digested with KpnI. All Ras constructs used in this manuscript were to the H-Ras isoform and are subsequently referred to only as Ras. pBABE-Ras constructs were a gift from Dr. Lawrence Quilliam (Indiana University, Indianapolis, IN). All Ras mutants were PCR amplified using the following primers, 5′CACCCGGGATCCTCAGGAGAGCACACAC3′ and 5′TGAGGATCCATG ACGGAATATAAG 3′ digested with BamHI and cloned into the BamHI site of pFlag-VN-173N. The RBD of Raf (aa 1–148) [24] was removed from pGEX with EcoRI, treated with Klenow fragment of DNA pol I, and then digested with BamHI. The Raf-RBD was then cloned into the EcoRV and BamHI sites of the pEFG vector. The RBD of PI3KC2β (aa 364–466) was PCR amplified from full-length PI3KC2β using the following primers: 5′ CGCAGATCTGCTGTCACCCCTAGC 3′, and 5′ CGCCCCGGGAACCTTCT GCTCCATCAGC 3′ digested with BglII and SmaI and then subcloned into YFP digested with BglII/SmaI. The pEFG-PI3KC2β-RBD was constructed by sequential digestion of the YFP-RBD construct with SmaI then BglII and cloning the resulting RBD fragment into pEFG SmaI and BamHI sites. The GST-PI3KC2β-RBD construct (aa 368–463) was constructed by PCR amplification using 5′ GAGAGAGACATATGAGCCCAGAGCA CCTCGGGGAT 3′ and 5′ GAGAGAGGATCCCTCCATCAGCTGTAGCCGAAT 3′. The resulting fragment was cloned into pcr4-TOPO (Invitrogen, Carlsbad, CA) and sequenced. The TOPO-PI3KC2β-RBD was digested with NdeI and BamHI, treated with the Klenow fragment of DNA pol I, then cloned into the SmaI site of pGEX-4T1. The Lys379Ala point mutation in the RBD of PI3KC2β was generated using 5′-CTCGGGGATGAGGTCAACCTGGCGGTGACTGTG-3′, 5′-CCTGTCACACAACACA GTCACCGCGAGGTTGAC3′. The Thr392Asp point mutation in the RBD of PI3KC2β was generated using, 5′-GACAGGCTTCAAGAGGCACTCGATTTCACCTGC-3′, 5′-GGAGGAACAGTTGCAGGTGAAATCGAGTGCCTC-3′, and cloned into CFP-PI3K as XhoI/BmgB1 fragments. CFP-PI3KC2β-K/A, and CFP-PI3KC2β-T/D were digested with EcoRI, treated with Klenow fragment of DNA pol I, then digested with KpnI. These fragments were then cloned into pHA-VC155N digested with XhoI, treated with Klenow fragment of DNA pol I, then digested with KpnI.

**Figure 5:**
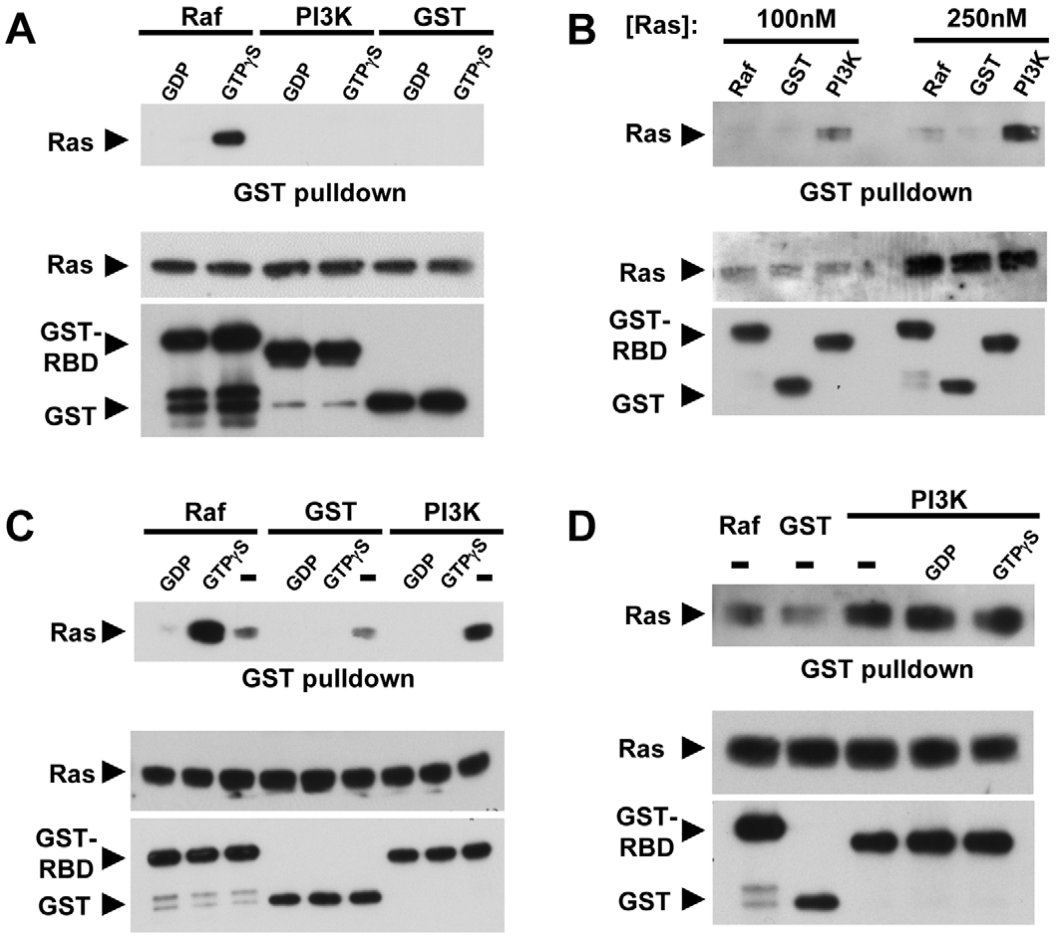
Binding of PI3KC2β-RBD to Ras. (A) Nucleotide loaded Ras does not directly interact with the RBD of PI3KC2β. Ras-GDP or Ras-GTPγS were incubated with GST-Raf-RBD, GST-PI3KC2β-RBD or GST alone as a negative control. Bound proteins were then analyzed by Western blot with a Ras antibody. The RBD of Raf specifically bound Ras-GTPγS. Neither GST-PI3KC2β-RBD nor GST alone interacted with Ras-GDP or Ras-GTPγS. Top panel, Ras bound to GST proteins. Bottom panels, input amounts of proteins. (B) Nucleotide-free Ras was generated in vitro as described and then tested for binding to the various GST proteins as in (A). GST-PI3KC2β-RBD directly binds nucleotide-free Ras while little association was seen with the GST-Raf-RBD or GST alone. Panels are same as in A. (C) Repeat of (B) except nucleotide (1 mM) was present during the binding reaction. Panels are same as in (A). (D) Addition of nucleotide (1 mM) does not disrupt pre-bound PI3KC2β-RBD- nucleotide-free Ras. GST-PI3KC2β-RBD was first bound to nucleotide-free Ras. Following binding, the complex was incubated with 1 mM GDP or GTPγS at RT for 30 min and then washed with buffer. Bound proteins were then analyzed as in (A).

### Protein purification

GST and GST-Raf-RBD were purified from DH5a cells. GST-PI3KC2β-RBD was purified from BL21 (DE3) cells. Bacterial cultures were grown overnight at 37°C. The following day bacterial cultures were diluted 1∶10, grown to an OD_600_ of 0.6, and induced with 0.1mM –0.2 mM IPTG for either 3 hrs at 37°C or overnight at room temperature. Cells were resuspended in MTPBS (16 mM Na_2_HPO_4_, 4 mM NaH_2_PO4-H_2_O, 150 mM NaCl, 50 mM EDTA, 1% TritonX-100, pH 7.3) and sonicated. The supernatant was then incubated with Glutathione Sepharose beads (GE Healthcare) for 1 hr at 4°C. Beads were then washed 3x with MTPBS and 3x with loading buffer (20 mM Tris-HCl pH 7.6, 10 mM EDTA, 5 mM MgCl_,_ 1 mM DTT, 0.1 mM PMSF, 10% glycerol). pQlink Ras 1–166 was a kind gift from Dr. Sharon Campbell (University of North Carolina, Chapel Hill, NC) and was used to express Ras in BL21 cells. Bacterial cultures were grown overnight at 37°C and the following day diluted 1∶50, grown to an OD_600_ of 0.6, and induced with 100 mM of IPTG overnight at room temperature. Bacteria were lysed in NiA buffer (50 mM HEPES, 150 mM NaCl, 5 mM MgCl_2_, 20 mM imidazole, 50 uM GDP, 100uM TCEP) and sonicated. The supernatant was incubated with nickel beads (Clontech, Mountain View, CA) for 30 min at room temperature, then washed with NiA and NiB buffer (50 mM HEPES, 150 mM NaCl, 5 mM MgCl_2_, 80 mM imidazole, 50 uM GDP, 100 uM TCEP). His-Ras was eluted off the nickel beads with NiC buffer (50 mM HEPES, 150 mM NaCl, 5 mM MgCl_2_, 300 mM imidazole, 50 uM GDP, 100 uM TCEP) and dialyzed ON at 4°C against Loading Buffer (20 mM Tris-HCl pH 7.6,10 mM EDTA, 5 mM MgCl_2_, 1 mM DTT, 0.1mM PMSF, 10% glycerol). The His tag was then cleaved off His-Ras with TEV protease (Promega) ON at 4°C. The TEV protease and uncleaved His-Ras was removed from the reaction by incubation with nickel beads.

**Figure 6:**
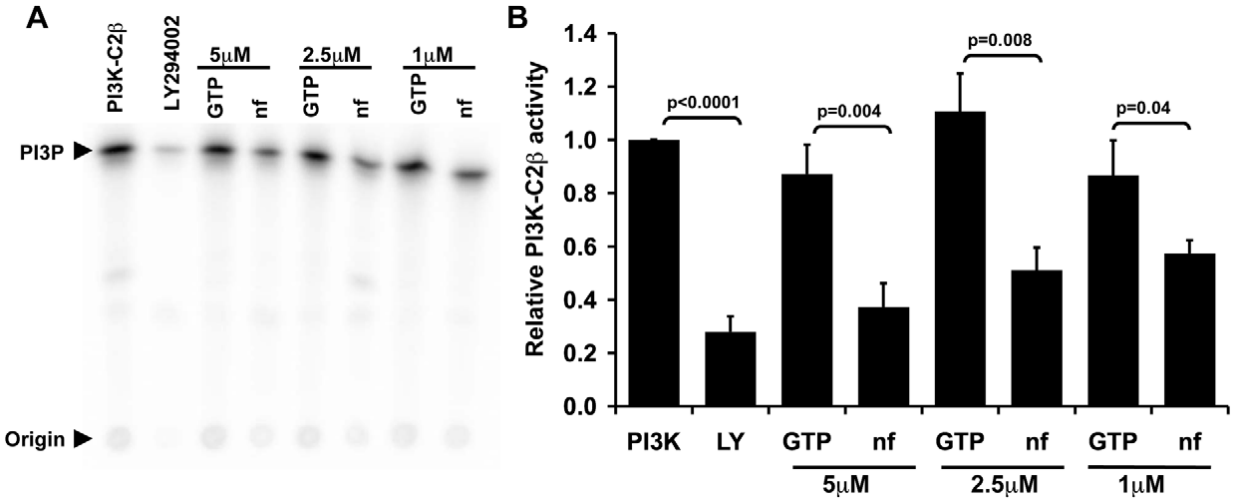
Nucleotide free Ras inhibits PI3KC2β activity. (A) The *in vitro* kinase activity of purified PI3KC2β was assayed in the presence of nucleotide-free (nf) Ras or Ras loaded with GTPγS (5 μM, 2.5 μM, and 1 μM). LY294002 (10 μM) was used as a negative control. The radioactive phospholipid products were extracted and eluted on a TLC plate. (B) Nucleotide-free Ras dose dependently inhibits PI3KC2β. LY294002 (10 μM; LY) inhibits PI3KC2β activity while Ras-GTP (5 μM, 2.5 μM, and 1 μM) does not. The graph represents relative kinase activity normalized to PI3KC2β alone ± S.E.M. from 4–6 independent experiments.

### In vitro pulldowns

Purified Ras was incubated in Loading Buffer (20 mM Tris-HCl pH 7.6, 15 mM EDTA, 5 mM MgCl_2_, 1 mM DTT, 0.1 mM PMSF, 10% glycerol) with either 1 mM GDP or 1 mM GTPγS for 30 min at 30°C. The reaction was stopped with 20 mM MgCl_2_ and kept on ice. 250 nM nucleotide loaded Ras was incubated with 850 nM GST-Raf-RBD, GST-PI3KC2β-RBD, or GST alone bound to glutathione beads for 1hr at 4°C. Beads were then washed 3x with Washing Buffer (10% glycerol, 5 mM MgCl_2_, 1 mM DTT, 0.1% TritonX-100, 0.1 mM PMSF) and run on an SDS-PAGE gel.

**Figure 7:**
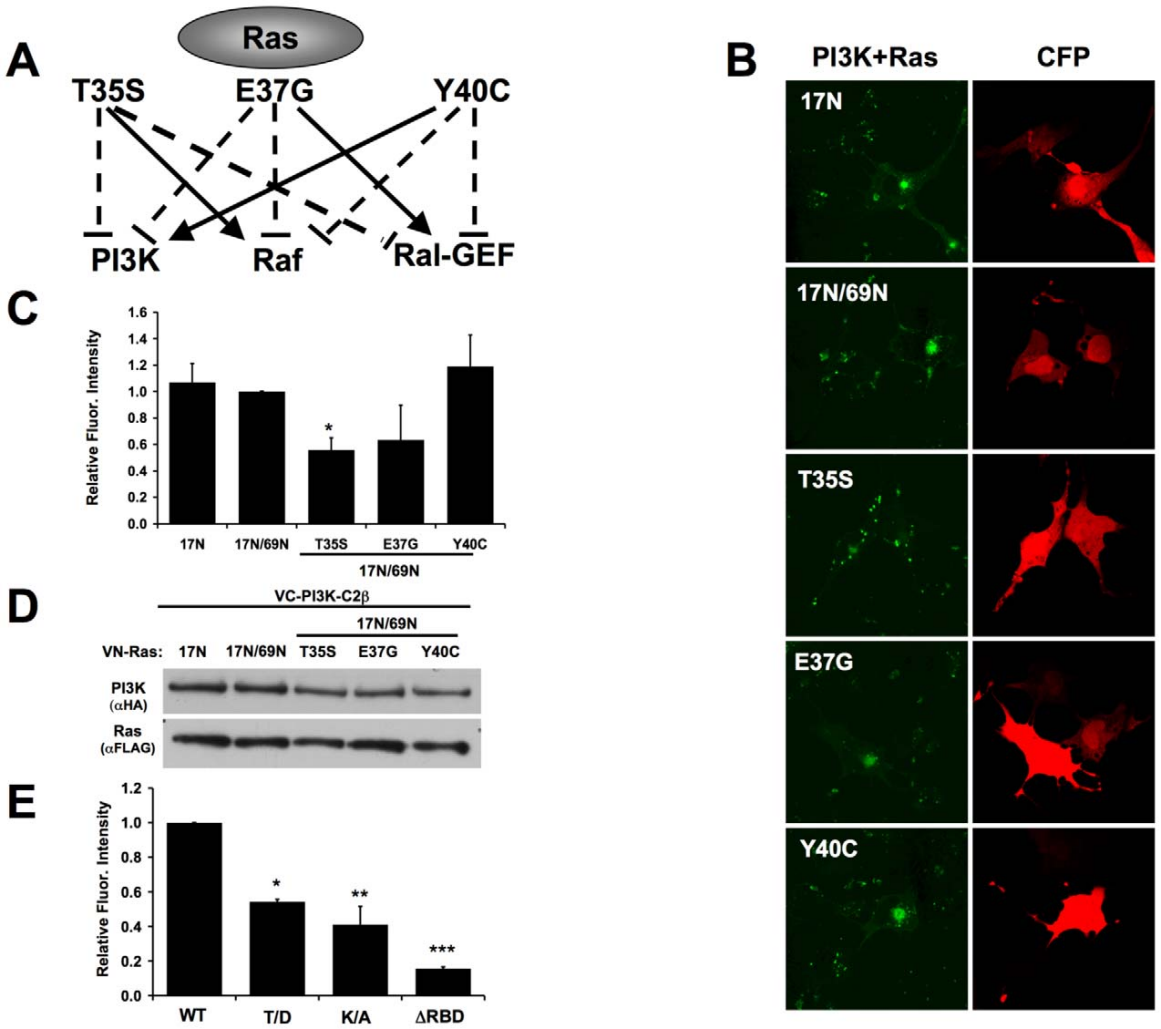
Mutations in the effector region of Ras disrupt PI3KC2β binding. (A) Point mutations in the effector region of Ras12V disrupt interactions with specific Ras targets. (B) VC-tagged PI3KC2β was co-transfected with either VN-tagged Ras17N, 17N/69N, or one the effector mutants in the background of Ras17N/69N. BiFC signal is pseudo-colored green. Effector mutations that disrupt Class I PI3K binding to Ras12V disrupt Class II PI3K binding to Ras17N/69N. CFP (red) was used as a transfection control. (C) Graph represents the average fluorescence intensity per cell ± S.E.M. from at least three independent experiments. (*p = 0.02). (D) Western blot analysis demonstrates equal expression of all constructs. (E) Mutation of Thr392 to Asp or Lys379 to Ala in full-length PI3KC2β disrupts interaction with Ras17N. The ΔRBD mutant was also included as a negative control. Graph represents the average of three independent experiments (*p<0.05).

### Nucleotide-free assays

Purified Ras 250 nM was incubated with either 850 nM GST-Raf-RBD, GST-PI3KC2β-RBD, or GST bound to Glutathione beads in Loading Buffer (20 mM Tris-HCl pH 7.6, 15 mM EDTA, 5 mM MgCl_2_, 1 mM DTT, 0.1 mM PMSF, 10% glycerol) for 30 min at 30°C in the absence of added nucleotide. Beads were then washed 3x with Loading Buffer. For the competition assays, 1 mM GDP or 1 mM GTPγS was added either directly to the binding reaction or to the third wash of the beads and incubated for 30 min at room temperature. Samples were then run on an SDS-PAGE gel.

**Figure 8:**
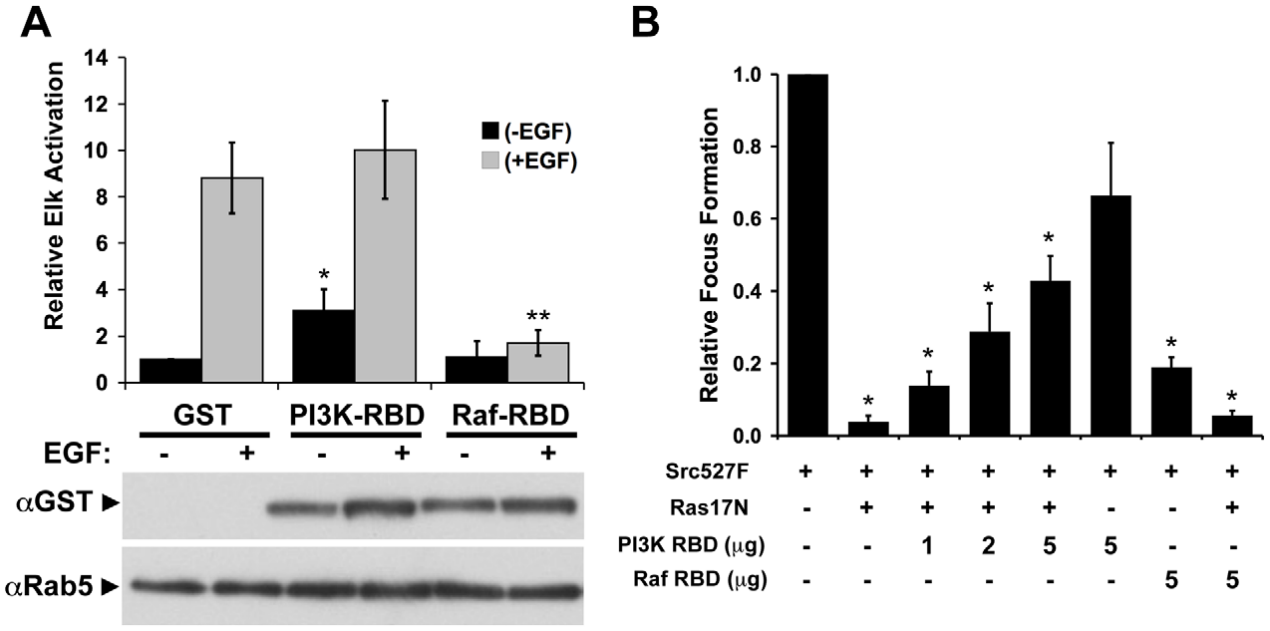
Biological activity of PI3KC2β versus Raf RBDs. (A) Expression of the Raf-RBD but not the PI3KC2β-RBD or GST alone inhibited EGF-stimulation of a Gal-Elk reporter assay [23]. GST-RBDs were expressed equally. GST alone migrated at a faster rate and was not visible in this image. Results represent the average relative activation ± S.E.M. from at least three independent experiments. (*p<0.05 compared to unstimulated GST, **p<0.01 compared to EGF stimulated GST). (B). PI3KC2β-RBD dose-dependently inhibits the effect of Ras17N on Src-mediated transformation. NIH/3T3 cells were transfected with 100 ng of SrcY527F expression construct in the presence or absence of Ras17N. Co-expression of the PI3KC2β-RBD reverses the inhibitory effect of Ras17N on Src transformation whereas the Raf-RBD does not. In contrast, expression of the Raf-RBD alone, but not the PI3KC2β-RBD, significantly inhibited Src-mediated transformation. The results represent the average relative focus forming activity ± S.E.M. from three independent experiments performed in triplicate. Asterisks denote samples that were significantly different from Src alone (*p<0.05).

### Biochemical analyses

Cells were treated and analyzed by Western blot as described previously [21]. BiFC, confocal imaging and analysis were performed as described [25]. Transient reporter assays using the Gal-Elk reporter were performed in HEK293T cells as previously described [23]. AKT activation assays were performed as in [22]. Briefly, COS cells were transiently co-transfected with the indicated expression constructs along with a HA-epitope tagged AKT expression construct. Following an overnight incubation in serum free media, cells were lysed in PLC-LB (50 mM HEPES, pH 7.5, 150 mM NaCl, 10%glycerol, 1% Triton X-100, 1 mM EGTA, 1.5 mM MgCl_2_ and 100 mM NaF) supplemented with protease inhibitors. Lysates were then analyzed by Western blots to assess the expression of HA-AKT and then normalized so that equal amounts of HA-AKT were immunoprecipitated with an HA monoclonal antibody. Precipitates were washed three times with ice cold PLC-LB containing inhibitors then resuspended in 25 μl of 4x NuPAGE sample buffer supplemented with 5%β ME. After heating to 70°C for 10 min, equivalent amounts of samples were fractionated on duplicate 4–12% NuPAGE gels (Invitrogen), transferred to Immobilon-P membranes and probed with antibodies to the HA epitope to determine total levels of AKT or antibodies to activated AKT (pSer473). Activation levels were determined by densitometric analysis of Western blots to determine pAKT and total AKT levels. Fold activation was determined by dividing the level of pAKT by total AKT and normalizing to unstimulated, vector control sample (YFP alone). Experiments were performed in triplicate.

**Figure 9:**
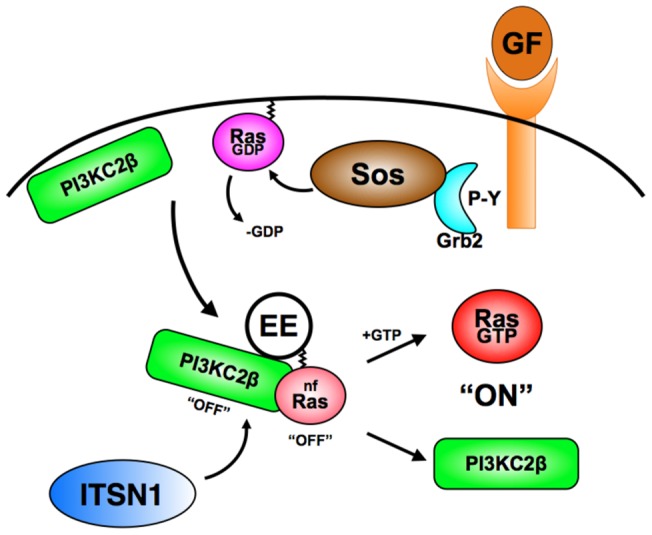
Model for ITSN1-Ras-PI3KC2β pathway. Growth factor stimulation of receptor tyrosine kinases leads to the recruitment of the Grb2-Sos complex resulting in dissociation of GDP from Ras. We propose that PI3KC2β competes for binding this transient nucleotide-free (nf) Ras trapping it in the nucleotide-free state and preventing GTP loading. In addition, binding of nucleotide-free Ras to PI3KC2β inhibits its lipid kinase activity. The PI3KC2β-Ras complex may then translocate to distal sites such as early endosomes (EE) where ITSN1 then binds to PI3KC2β leading to the release of nucleotide-free Ras and activation of the lipid kinase activity of PI3KC2β. In addition, once released from the ITSN1-PI3KC2β complex, Ras binds GTP resulting in Ras activation and recruitment of effectors, e.g., Raf, Class I PI3Ks, etc. Although the above model describes a potential role for nf-Ras in ITSN1 and PI3KC2β function, we propose that nf-Ras may be regulated by additional proteins besides these molecules. Our findings also raise the possibility that the nucleotide-free forms of other GTPases may play a similar role in cell signaling.

### In vitro kinase assays

To measure the effect of Ras on PI3KC2β activity, pulldowns with Ras were performed as described above, but using full length GST-PI3KC2β (Invitrogen, Carlsbad, CA). 2x Kinase Buffer (40 mM Tris-HCl, 200 mM NaCl, 2 mM DTT) was added to GST-PI3KC2β: Ras beads. The reaction was started by addition of ATP (40 uM), phosphatidylinositol (0.2 mg/mL), MgCl_2_ (3.5 mM), and [γ-^32^P]-ATP (10 mCi). Samples were incubated for 15 min at 37°C. 100 mL of 1 N HCl was added to stop the reaction, followed by 200 mL of CHCl_3_: MeOH (1∶1) to extract the lipid. The organic phase was collected and a second extraction was performed by adding 70 mL of MeOH: 1N HCl (1∶1). The organic phase was collected, the samples dried, re-suspend in 30 mL of CHCl_3_: MeOH (1∶1), and spotted onto a TLC (Whatmann, Kent, ME) plate that had been pre-treated with 1% oxalic acid, 1 mM EDTA, 40%MeOH, and baked at 110°C for 1 hr. Plates were developed in 34 mL water, 65 mL n-propanol, and 1 mL glacial acetic acid. Phospho-images were developed on a STORM 860 phosphoimager (GE Healthcare) and quantified using ImageQuant 5.2 (Molecular dynamics).

## Results

### PI3KC2β and Ras interact and co-localize with ITSN1 on intracellular vesicles

Co-localization of PI3KC2β to ITSN1-positive vesicles [22] coupled with ITSN1 activation of Ras on similar vesicles [24] and the presence of a putative RBD on PI3KC2β [22] prompted us to examine whether PI3KC2β was a target of the ITSN1-Ras pathway. Although a previous report suggested that PI3KC2β and Ras do not interact *in vitro* [26], we sought to determine whether PI3KC2β interacted with Ras in cells using bimolecular fluorescence complementation (BiFC) [27], [28]. We observed significant interaction between PI3KC2β and Ras on a perinuclear population vesicles (Fig. 1A). The BiFC complex of Ras and PI3KC2β co-localized with CFP-ITSN1 (Fig. 1A) suggesting that all three molecules are indeed in a trimolecular complex. This pattern of fluorescence was reminiscent of the localization of ITSN1-Ras as visualized by FRET [24] and ITSN1-PI3KC2β as visualized by co-localization and BiFC [22]. Ras interaction with PI3KC2β required the RBD as deletion of this region significantly reduced the BiFC signal (Fig. 1B). Although mutation of the PRD does not significantly reduce Ras-PI3KC2β interaction, ITSN1 association with PI3KC2β is abolished [22]. Interestingly, deletion of the RBD also reduces ITSN1 interaction with PI3KC2β suggesting that Ras is important for ITSN1-PI3KC2β association (data not shown).

### Ras is necessary for ITSN1 activation of AKT

PI3K activity is necessary for ITSN1 activation of AKT and for regulation of an ITSN1 survival pathway in neuronal cells [22]. Given the co-localization of ITSN1, Ras, and PI3KC2β, we tested the importance of Ras in ITSN1 activation of AKT. Expression of dominant-negative Ras (Ras17N) inhibited ITSN1 activation of AKT (Fig. 2). Since Ras17N acts as a dominant-negative by sequestering Ras GEFs and preventing nucleotide dissociation from endogenous Ras [9], we tested whether this inhibition of AKT activation was due entirely to inhibition of Ras interaction with exchange factors. Mutation of Asp 69 to Asn (69N) disrupts interaction of Ras with GEFs such as Sos and RasGRF [11]. Expression of Ras17N/69N inhibits ITSN1 activation of AKT suggesting that inhibition of this pathway is independent of blocking Ras activation by GEFs (Fig. 2).

### PI3KC2β forms a complex with Ras on intra-cellular vesicles

The presence of an RBD on PI3KC2β and the co-localization of Ras with PI3KC2β suggest that PI3KC2β is a *bona fide* Ras effector. Surprisingly, constitutively activated Ras, Ras61L, exhibited little interaction with PI3KC2β (Fig. 3A). Similar results were observed with Ras12V (Fig. 3B). In contrast, Ras17N interacted most strongly with PI3KC2β followed by RasWT (Fig. 3). The interaction between Ras17N and PI3KC2β was not dependent on exchange factors since this interaction with Ras17N/69N was only moderately reduced when compared to interaction with Ras17N (Fig. 3A). In contrast, ITSN1 interaction with Ras17N was dramatically reduced by the 69N mutation consistent with the finding that ITSN1 interacts with Ras through binding Ras GEFs (Fig. 4) [29]. Mutation of the ITSN1-binding site in PI3KC2β did not significantly affect Ras-PI3KC2β interaction (Fig. 1B) further supporting the conclusion that exchange factor binding (through ITSN1) is not necessary for Ras-PI3KC2β interaction. However, deletion of the RBD dramatically reduced Ras-PI3KC2β interaction (Fig. 1B).

### The PI3KC2β RBD directly interacts with nucleotide-free Ras

While Ras17N is reported to bind GDP in cells [12], *in vitro* studies indicate that Ras17N binds nucleotides with a ∼30-fold reduced affinity compared to wild-type Ras suggesting that it exists in the nucleotide-free state for extended periods *in vivo* [9]. Thus, the interaction between PI3KC2β and Ras17N raises the possibility that the PI3KC2β-RBD interacts with either RasGDP or nucleotide-free Ras. To determine the nucleotide dependence for Ras-PI3KC2β binding, we examined the interaction of Ras and PI3KC2β *in vitro* using purified proteins. Bacterially expressed and purified Ras (aa 1–166) was loaded with nucleotide (GDP or GTPγS) and tested for interaction with GST, GST-Raf-RBD (aa 1–148), or GST-PI3KC2β-RBD (aa 368–463). As expected, GST-Raf-RBD bound preferentially to RasGTPγS [30]. In contrast, neither GST-PI3KC2β-RBD or GST interacted with nucleotide-bound Ras (Fig. 5A), consistent with prior findings from Waterfield and colleagues [26]. To determine if the PI3KC2β-RBD interacts with nucleotide-free Ras, we generated nucleotide-free Ras *in vitro* (see Materials and Methods). GST-PI3KC2β-RBD directly bound nucleotide-free Ras, while little binding was observed between GST-Raf-RBD or GST alone (Fig. 5B). Addition of nucleotide (either GDP or GTPγS) to the binding reaction prior to incubation at 30°C inhibited interaction of Ras and GST-PI3KC2β-RBD. Addition of GTPγS to the GST-Raf-RBD binding reaction, however, induced Ras-Raf interaction (Fig. 5C). These results indicate that under these experimental conditions, nucleotide-free Ras is generated and in the presence of nucleotide, Ras becomes nucleotide loaded. Next, we examined whether nucleotide could compete off Ras once bound to the PI3KC2β-RBD, a function previously observed with the Ras GEF, CDC25 [31]. Addition of 1 mM nucleotide, either GTP or GDP, was not sufficient to disrupt the complex of Ras-PI3KC2β-RBD (Fig. 5D). These results suggest that PI3KC2β directly interacts with nucleotide-free Ras, conceivably stabilizing this nucleotide-free intermediate.

### Nucleotide-free Ras inhibits PI3KC2β activity

Ras17N inhibits PI3K-dependent ITSN1 activation of AKT (Fig. 2), suggesting that nucleotide-free Ras may negatively regulate PI3KC2β activity. To determine the effect of nucleotide-free Ras on PI3KC2β activity, *in vitro* PI3KC2β activity was measured in the presence of nucleotide-free or GTP-loaded Ras (Fig. 6). Treatment with LY294002 (10 uM) inhibits *in vitro* PI3KC2β activity as expected. Interestingly, addition of nucleotide-free Ras, but not RasGTPγS, dose dependently inhibits PI3KC2β activity.

### Point mutations in the effector domain of Ras or the RBD of PI3KC2β disrupt Ras-PI3KC2β interaction

Effectors of activated Ras bind the switch 1 region of Ras (aa 25–45), also termed the effector domain. Point mutations (Thr35Ser, Glu37Gly, and Tyr40Cys) in this region of activated Ras (Ras12V) differentially disrupt the interaction with specific effectors (Fig. 7A) [32]. We examined whether these mutations in the context of Ras17N/69N disrupted binding to PI3KC2β. Ras17N/69N was chosen over Ras17N to avoid any potential complications from Ras interaction with exchange factors. Mutation of Thr35 and Glu37 of Ras17N/69N decreased PI3KC2β binding whereas Tyr40 mutation had no appreciable effect (Fig. 7B–D). These mutations in the context of activated Ras (Ras12V) have a similar effect on interaction with Class I PI3Ks [32] suggesting that PI3KC2β interacts with the same region of Ras as Class I PI3Ks but only when Ras is nucleotide-free. Conversely, specific mutations in the RBD of Class I PI3Ks (Thr208 of PI3K-C1α) block Ras binding both *in vitro* and *in vivo* [33], [34]. Although the RBD of PI3KC2β shares only 18% identity with PI3K-C1α ΔRBD, mutation of the analogous Thr in PI3KC2β to Asp (Thr392 to Asp) resulted in a significant decrease in Ras binding (Fig. 7E). Furthermore, based on homology modeling of the PI3KC2β RBD onto the structure of the PI3K-C1γ RBD, Lys379 of the PI3KC2β-RBD may also influence interaction with Ras. Indeed, mutation of Lys379 to Ala in the PI3KC2β-RBD resulted in significant impairment in Ras binding (Fig. 7E). These results indicate that the PI3KC2β-RBD utilizes a similar molecular surface as Class 1 PI3K RBD in the recognition of the switch 1 region of Ras. However, PI3KC2β binding is specific to nucleotide-free Ras and must be mediated by distinct structural differences.

### PI3KC2β alters Ras signaling

Our results predict that the RBD of Raf, but not PI3KC2β, would block Ras-dependent signaling. Indeed, expression of the Raf-RBD dramatically decreased Elk-1-dependent transcription by >80% whereas PI3KC2β-RBD expressing cells were not inhibited (Fig. 8A) further supporting the model that the PI3KC2β-RBD does not interact with Ras-GTP.

To determine whether the PI3KC2β-RBD is active in binding targets *in vivo*, we tested the ability of the PI3KC2β-RBD to block the inhibitory activity of Ras17N (Fig. 8B). The transforming activity of oncogenic Src (SrcY527F) was reversed in the presence of Ras17N consistent with the requirement for Ras in Src-mediated transformation [10], [35]. Expression of PI3KC2β-RBD dose-dependently blocked Ras17N inhibition of Src transformation consistent with the binding of nucleotide-free Ras by PI3KC2β-RBD (Fig. 5). In contrast, expression of the Raf-RBD did not affect Ras17N inhibition of Src transformation. However, like Ras17N, expression of only the Raf-RBD with oncogenic Src inhibited transformation consistent with the role of active Ras in Src transformation [10], [35] and with the ability of this RBD to bind and inhibit activated Ras. In contrast, expression of the PI3KC2β-RBD alone did not inhibit Src transformation consistent with its inability to bind RasGTP (Fig. 8B).

## Discussion

Ras, like all GTPases, cycles between an inactive GDP-bound state and an active GTP-bound state. The transition from the inactive to active state requires formation of nucleotide-free Ras through the action of exchange factors. This state is considered to be a short-lived transition state intermediate *in vivo* [36] based on the relatively high GTP: GDP ratio *in vivo* [37], the ability of GTP to dissociate the GEF-Ras complex *in vitro* [31], and the assumption that there are no proteins *in vivo* that might stabilize nucleotide-free Ras and prevent GTP loading. However, our results provide the first direct evidence for a protein that may stabilize nucleotide-free Ras *in vivo*. We demonstrate that the RBD of PI3KC2β binds nucleotide-free Ras *in vitro* (Fig. 5). In contrast to the GEF-Ras complex, which is disrupted by addition of guanine nucleotides, the PI3KC2β RBD-Ras complex is stable even in the presence of high concentrations of GTP or GDP. These data suggest that PI3KC2β binding to nucleotide-free Ras *in vivo* may prevent loading of nucleotides onto Ras. Although current methods do not allow for detection of nucleotide-free GTPases *in vivo*, our BiFC results provide additional support for our model. PI3KC2β preferentially interacts with Ras17N, which has a 30-fold lower affinity for nucleotide compared to wild type Ras and therefore should exist for longer periods in the nucleotide-free state. As a result, BiFC traps this form of Ras resulting in greater fluorescence complementation for Ras17N (and Ras17N/69N) compared to wild type or constitutively activated Ras (61L or 12V).

Our findings also reveal a biochemical role for nucleotide-free Ras in regulating cell signaling. Addition of nucleotide-free Ras to PI3KC2β inhibited its *in vitro* lipid kinase activity compared to addition of RasGTPγS. This result is consistent with our observations that ITSN1 activation of AKT is blocked by expression of Ras17N and Ras17N/69N, which is impaired in GEF binding (Fig. 2), and dependent on PI3K activity [22]. Thus, PI3KC2β represents the first identified biochemical target of nucleotide-free Ras. Our results challenge the long held assumption that nucleotide-free Ras is a short-lived intermediate *in vivo* in the generation of RasGTP [36]. However, proof of the presence of nucleotide-free Ras bound to PI3KC2β, or potentially other targets, *in vivo* still awaits formal demonstration.

Based on these observations, we propose that once nucleotide-free Ras is generated through GEF-stimulated nucleotide release, PI3KC2β binding, perhaps in conjunction with additional factors, displaces the GEF and stabilizes nucleotide-free Ras (Fig. 9). This interaction has two potential consequences: inhibition of GTP loading on Ras bound to PI3KC2β and inhibition of PI3KC2β activity. Such a mechanism might allow for the transport of nucleotide-free Ras to distal cellular compartments where PI3KC2β dissociates from Ras upon some trigger, e.g., ITSN1 binding to PI3KC2β. The dissociation of nucleotide-free Ras from PI3KC2β would then lead to immediate GTP loading, i.e., Ras activation, as well as derepression of PI3KC2β. Indeed, the PI3KC2β-RBD-Ras complex is refractory to dissociation by high nucleotide concentrations (Fig. 5D) suggesting that additional factors are necessary for this dissociation. Since ITSN1 activates both Ras and PI3KC2β [22], [24], we propose that ITSN1 represents one such factor. Furthermore, RasGTP could then couple to specific Ras effectors at these sites leading to compartmentalized activation of these targets. While there are clear examples for compartmentalized activation of Ras at intracellular sites by specific GEFs [38], our model suggests a GEF-independent activation of Ras at such intracellular sites. Such a mechanism would decouple GEF localization from GTPase activation while allowing for integration of multiple signaling pathways.

Our results also suggest an added level of complexity to Ras signaling and transformation. Most efforts at understanding Ras-mediated transformation have centered on identifying those targets that bind RasGTP. However, our data raise the possibility that there is a class of proteins, such as PI3KC2β, that bind nucleotide-free Ras and are negatively regulated by this interaction. Indeed, wild type Ras antagonized Ras-mediated transformation, consistent with this possibility [2], [3], [4], [5]. Oncogenic activation of Ras would lead to loss of this negative regulation resulting in activation of these targets. Such targets may contribute to Ras-induced transformation without binding activated Ras. Indeed, we have recently implicated PI3KC2β in neuroblastoma tumorigenesis [39].

It has been long accepted that Class I PI3Ks are effectors of Ras-GTP. However, our data demonstrates for the first time a role for Ras in Class II PI3K regulation. Point mutations in the effector region of Ras that disrupt Class I PI3K binding also disrupt Class II PI3K binding. In addition, concomitant point mutations in the RBDs of Class I and Class II PI3Ks disrupt Ras association (Fig. 7). Taken together these data suggest that Class I and Class II PI3Ks associate with the same region of Ras, but that this association is mediated by distinct structural differences in the PI3Ks as well as Ras-GTP vs nucleotide-free Ras.

In addition to PI3Ks, many other proteins have been identified that contain RBDs or Ras-Association (RA) domains [40]. Although, these domains have little sequence homology, they adopt a similar ubiquitin-fold structure [41]. Interestingly, a group of these domains including Myr5, Tiam-1, Rain, and PLCε do not bind nucleotide-loaded Ras raising the question of whether additional RBDs may share a similar activity in binding nucleotide-free Ras [41], [42], [43]. In addition, Class II PI3K alpha (PI3KC2α) also contains an RBD that is 53% similar to PI3KC2β-RBD warranting further investigation into the Ras-binding properties of this PI3K isoform.

While RasGDP and RasGTP are thought of as the predominant forms of Ras involved in cell signaling, our findings raise the possibility that nucleotide-free Ras may also function in regulating cellular signaling *in vivo*. Our results demonstrate that *in vitro*, nucleotide-free Ras binds PI3KC2β and that this complex is refractory to dissociation by guanine nucleotides. Furthermore, nucleotide-free Ras inhibits the lipid kinase activity of PI3KC2β suggesting that nucleotide-free Ras plays a negative regulatory role in signaling. Our findings suggest a novel model for compartmentalized signaling in which PI3KC2β results in the redistribution of nucleotide-free Ras to intracellular vesicles leading to localized Ras activation and engagement of specific Ras effectors resulting compartmentalized activation of these targets. While our studies have been limited to H-Ras, both N- and K-Ras isoforms exhibit compartmentalized signaling activity as well [38], [44], [45] suggesting that the nucleotide-free forms of these Ras isoforms may also play an active role in regulating signaling. Our results raise the further possibility that nf-forms of other Ras-related GTPases may play an unappreciated role in cell signaling. Due to the broad role of Ras in cell growth, development, and oncogenesis, our findings suggest that the role of nucleotide-free Ras in cell signaling warrants further examination.
